# Kyphotic Posture Reduces Respiratory Efficiency During Walking and Running

**DOI:** 10.7759/cureus.93367

**Published:** 2025-09-27

**Authors:** Noboru Chiba, Tadayoshi Minamisawa, Yuka Matsuda, Toshihiko Fujimoto

**Affiliations:** 1 Department of Occupational Therapy, Yamagata Prefectural University of Health Sciences, Yamagata, JPN; 2 Department of Physical Therapy, Yamagata Prefectural University of Health Sciences, Yamagata, JPN; 3 Department of Occupational Therapy, Minamisagae Hospital, Sagae, JPN; 4 Institute for Excellence in Higher Education, Tohoku University, Sendai, JPN

**Keywords:** end-tidal co₂, kyphotic posture, respiratory exchange ratio, vd/vt, ventilatory efficiency

## Abstract

Background: Altered spinal alignment can affect ventilatory mechanics, yet the acute impact of kyphotic posture during dynamic exercise remains unclear. This study aimed to examine the acute effects of a simulated kyphotic posture on ventilatory efficiency during treadmill standing, walking, and running in healthy young adults. We hypothesized that kyphotic posture would increase ventilatory burden, particularly at higher exercise intensities.

Methods: Ten healthy young men completed two postural conditions (kyphotic and neutral) while standing, walking (4 km/h), and running (10 km/h) on a rehabilitation treadmill (Biodex Medical Systems, Inc., Shirley, NY, USA). Spinal curvature was quantified using three-dimensional motion capture (VICON Motion Systems Ltd., Oxford, UK; 50 Hz) as the spinal curvature angle (θ) defined by the C7-PSIS-midpoint and C7-T10 lines. Breath-by-breath gas exchange was measured using Aeromonitor AE-310s (Minato Medical Science Co., Ltd., Osaka, Japan) under controlled ambient conditions. The variables included oxygen uptake (VO₂), carbon dioxide output (VCO₂), mass-specific oxygen uptake (VO₂/W), respiratory exchange ratio (RER), respiratory rate (RR), minute ventilation (VE), end-tidal oxygen (ETO₂), end-tidal CO₂ partial pressure (ETCO₂), VE/VO₂, VE/VCO₂, and physiological dead space to tidal volume ratio (VD/VT). Steady-state values were extracted from the final 1-min epoch of each condition (<5% coefficient of variation for VO₂ over 30 s). Paired t-tests or Wilcoxon signed-rank tests were used as appropriate; effect sizes were reported as dz (t-tests) or rrb (Wilcoxon) with 95% confidence intervals (CIs) (α=0.05).

Results: Kyphotic posture significantly increased spinal flexion during all tasks. At rest, the kyphotic posture showed a higher RR and ETCO₂, lower ETO₂, and higher VD/VT than the neutral posture (all p≤0.05). During walking (4 km/h), the RER was higher in the kyphotic posture (p=0.049), and ETCO₂ remained elevated; VO₂ and VO₂/W did not differ, whereas VCO₂ and VE/VCO₂ showed non-significant trends. During running (10 km/h), the kyphotic posture produced higher VCO₂ (p=0.036) and RER (p=0.004), with a non-significant trend toward higher VE; VO₂ and VO₂/W remained comparable between the postures. Across conditions, several effects were supported by confidence intervals consistent with posture-related differences, despite the modest sample size of the study.

Conclusions: A transient, simulated kyphotic posture was associated with a small but consistent increase in ventilatory burden during dynamic exercise in healthy young men, reflected by higher ETCO₂ at rest, higher RER during walking and running, and higher VCO₂ during running, despite similar VO₂. These preliminary findings warrant confirmation in larger, sex- and age-diverse cohorts, including individuals with structural kyphosis.

## Introduction

Running performance depends not only on cardiovascular capacity and muscular strength but also on respiratory system efficiency. Posture is a critical determinant of respiratory mechanics, as thoracic expansion, rib cage mobility, and diaphragm excursion are influenced by spinal alignment. Among athletes, coaches, and clinicians, it is widely believed that suboptimal running posture, particularly excessive forward trunk inclination or thoracic kyphotic posture, impairs breathing efficiency and, consequently, athletic performance [1,2]. Despite this prevailing assumption, there is a paucity of experimental studies that have quantitatively evaluated this effect during dynamic activities such as running.

This knowledge gap has important clinical implications. Individuals with pre-existing spinal deformities, such as thoracic kyphosis, scoliosis, or postural changes secondary to disability, may experience amplified ventilatory limitations during running. Furthermore, fatigue-induced forward trunk inclination is common in long-distance running, potentially accelerating the decline in respiratory efficiency and contributing to premature fatigue [3,4]. Understanding the biomechanical and physiological mechanisms underlying these changes is essential for developing targeted interventions that preserve performance and minimize respiratory strain.

Previous investigations have primarily focused on the effects of static kyphotic posture on pulmonary function in standing or seated positions and the benefits of postural correction in clinical populations [5-7]. Interventions aimed at improving thoracic mobility, such as thoracic spinal manipulation, have also been shown to enhance pulmonary function in sedentary young adults [5], reinforcing the link between spinal alignment and respiratory function. However, the interaction between spinal alignment and respiratory efficiency while running remains largely unexplored. Addressing this gap could inform competitive sports performance optimization and rehabilitation strategies for individuals with compromised postures.

Posture plays a critical role in respiratory mechanics, and deviations, such as kyphotic posture, can impair breathing efficiency. This may reduce athletic performance and exacerbate respiratory difficulties in individuals with such disabilities.

Kyphotic posture has been linked to reduced pulmonary function in elderly and patient populations. However, early identification of its acute effects in healthy adults may enable the implementation of preventive strategies before irreversible structural changes occur [5-7].

Therefore, we aimed to examine the acute effects of a simulated kyphotic posture on ventilatory efficiency during treadmill exercise (standing, walking at 4 km/h, and running at 10 km/h) in healthy young men. We hypothesized that kyphotic posture would increase ventilatory burden, particularly at higher exercise intensities. These findings may ultimately inform posture-focused interventions to optimize athletic performance and support respiratory rehabilitation in clinical populations.

## Materials and methods

All trials were conducted on a rehabilitation treadmill (Biodex Medical Systems, Inc., Shirley, NY, USA) set to 0% grade under constant-velocity control. Participants wore their own lace-up running shoes with rubber outsoles. After sufficient warm-up, each posture condition comprised three activities performed consecutively in the following order: standing at rest, walking (target speed 4 km/h), and running (target speed 10 km/h). Speed was gradually increased to the target for each activity; once the speed was constant, participants maintained that speed for 2 min. Handrail use was not permitted except during transitions for safety.

**Figure 1:**
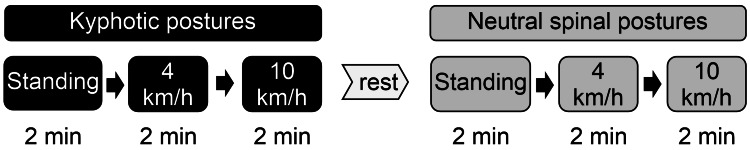
Experimental protocol for posture-respiration evaluation Schematic representation of the experimental procedure. The participants performed three activities —standing at rest, walking at 4 km/h, and running at 10 km/h—under both simulated kyphotic and neutral spinal postures. Each postural condition was preceded by adequate rest until the heart rate returned to baseline. A rest period of at least one hour was provided between the two posture trials to minimize carryover effects. Respiratory data were recorded during the final steady-state minute after confirming stabilization of oxygen uptake.

Postural conditions were administered in a fixed sequence (kyphotic first, then neutral). Consistent with Figure 1, a rest interval of at least 1 h was provided between posture blocks, and each block began only after confirmation that heart rate had returned to baseline (within ±5 bpm for ≥60 s; Polar V800 with chest strap).

Kyphotic posture

Participants voluntarily adopted a self-simulated kyphotic posture, characterized by thoracic flexion, forward head position, and internal shoulder rotation.

Neutral posture

Participants wore a postural support belt (MobiBelt, F-Assist Co., Sendai, Japan) designed to disengage when poor posture occurred, thereby facilitating the maintenance of proper alignment.

To enhance reproducibility, participants received standardized postural instructions and completed practice trials (including familiarization with the support belt) before data collection. The experimental protocol, including rest intervals, measurement order, and postural conditions, is summarized in Figure 1.

Data acquisition

Posture Analysis

**Figure 2:**
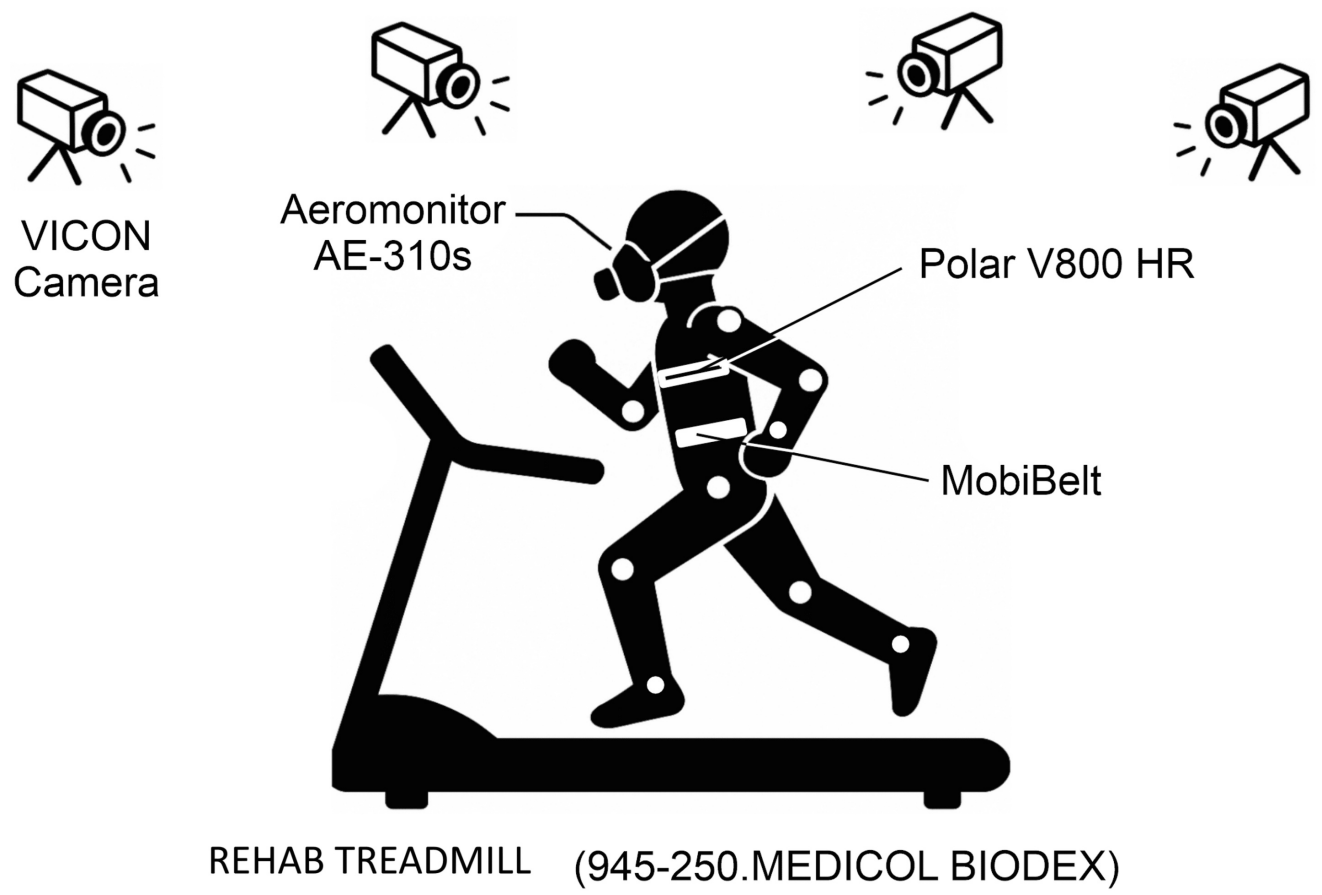
Experimental setup for assessing respiratory and biomechanical parameters during treadmill exercise This schematic illustrates the experimental setup used to evaluate the respiratory efficiency and body kinematics during treadmill locomotion. The participant performed walking or running on a rehabilitation treadmill, while eight VICON cameras (MX T20, VICON Motion Systems Ltd., Oxford, UK) captured the 3D motion data. Respiratory parameters were monitored using the Aeromonitor AE-310s mask system (Minato Medical Science Co., Ltd., Osaka, Japan). Heart rate was monitored using a Polar V800 HR with a chest strap, and during the neutral posture, a postural support belt was worn around the abdomen to maintain alignment while postural data were collected. The combined system enables the synchronized collection of respiratory, cardiovascular, and kinematic data at different exercise intensities in a single test.

The experimental setup is illustrated in Figure 2, which includes treadmill locomotion with simultaneous motion capture and respiratory gas analysis in both postures. Spinal alignment was assessed using a three-dimensional motion capture system (VICON Motion Systems Ltd., Oxford, UK; 50 Hz). Thirty-five reflective markers were placed according to the Plug-in-Gait full-body model. Spinal curvature was quantified as the spinal curvature angle (θ), defined as the angle formed between (1) a line connecting the seventh cervical vertebra (C7) to the midpoint between the left and right posterior superior iliac spines (PSISs), and (2) a line connecting C7 to the tenth thoracic vertebra (T10).

**Figure 3:**
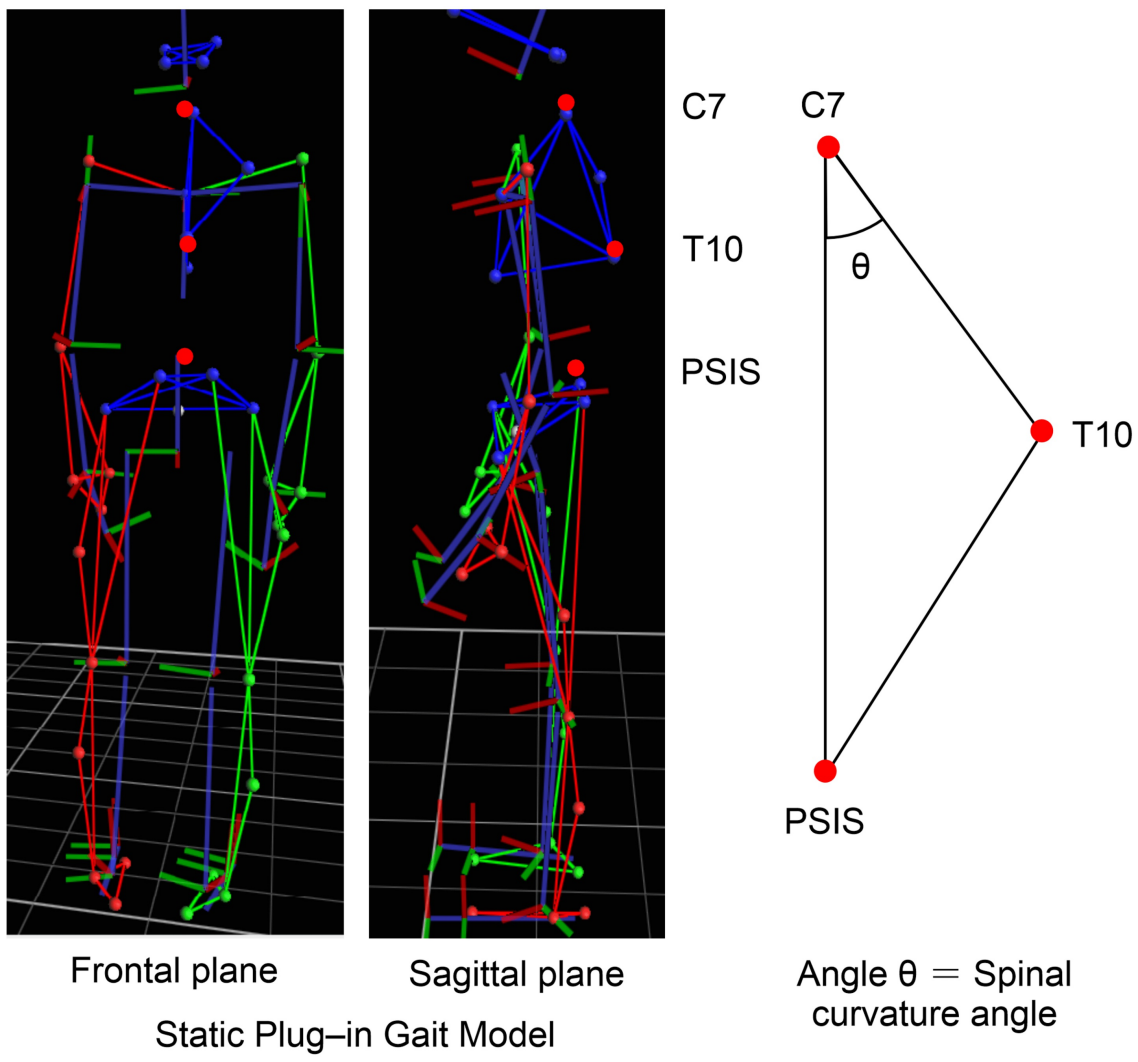
Measurement of spinal curvature angle (θ) using 3D motion capture The spinal curvature angle (θ) was defined based on the sagittal alignment of three anatomical landmarks: the seventh cervical vertebra (C7), the tenth thoracic vertebra (T10), and the midpoint between the bilateral posterior superior iliac spines (PSISs). The angle was calculated from the lines connecting C7–T10 and C7–PSIS in the sagittal plane using the Plug-in-Gait full-body marker set and the VICON motion analysis system.

As shown in Figure 3, this measurement captures the sagittal spinal alignment using the anatomical landmarks C7, T10, and PSIS midpoint. This method has been validated in previous studies [8,9] on dynamic postural assessment.

Respiratory Analysis

Breath-by-breath measurements were obtained using the Aeromonitor AE-310s (Minato Medical Science Co., Ltd., Osaka, Japan) under controlled ambient conditions (22-24°C, 50-60% RH). The variables included oxygen uptake (VO₂), mass-specific oxygen uptake (VO₂/W, mL·kg⁻¹·min⁻¹), carbon dioxide output (VCO₂, mL/min), respiratory exchange ratio (RER), respiratory rate (RR, breaths/min), minute ventilation (VE, L/min), end-tidal O₂ (ETO₂, kPa), end-tidal CO₂ partial pressure (ETCO₂, kPa), ventilatory equivalents (VE/VO₂, VE/VCO₂), and physiological dead space-to-tidal volume ratio (VD/VT). Steady-state values were extracted from the final 1-min epoch of each condition with a stable VO₂ (<5% coefficient of variation (CV) over 30 s).

Statistical analysis

For spinal curvature angles, framewise values were first averaged within each trial and then within each participant to yield one representative value per condition (Neutral, Kyphotic). The normality of the paired difference (Kyphotic-Neutral) was assessed using the Shapiro-Wilk test. If normality was observed, conditions were compared using the paired t-test; otherwise, the Wilcoxon signed-rank test was used.

For the respiratory variables, the final 60 s of each condition (steady-state VO₂) were averaged to obtain participant-level values, and the same inferential procedures were applied. Results are reported as mean ± SD for normally distributed variables and as median (Q1-Q3) for non-normally distributed variables. Effect sizes were expressed as Cohen’s dz for paired t-tests and rank-biserial correlation (rrb) for Wilcoxon tests, along with 95% confidence intervals for the mean/median differences. The significance threshold was set at p < 0.05 (two-tailed). Analyses were conducted using IBM SPSS Statistics for Windows, Version 26 (Released 2019; IBM Corp., Armonk, New York, United States).

## Results

Estimated VO₂ max and exercise intensity

The estimated VO₂ max was 41.2 ± 9.0 mL/kg/min. During treadmill walking at 4 km/h, VO₂/W was 12.5 ± 1.4 mL/kg/min, and running at 10 km/h elicited 33.9 ± 2.5 mL/kg/min, indicating low and high metabolic demands, respectively, under the neutral posture condition.

Postural metrics

As shown in Table 1, spinal curvature angles were significantly greater in the kyphotic posture across all activity conditions. In the standing position, the kyphotic angle was 16.30 ± 5.0°, compared with 6.0 ± 2.0° in the neutral posture (p < 0.001, Cohen’s dz = 2.02), with a mean difference of +10.3° (95% confidence interval (CI): 6.6-13.9). At 4 km/h, the kyphotic posture exhibited significantly greater thoracolumbar flexion than the neutral posture (14.0 ± 2.1° vs. 6.4 ± 2.1°, p < 0.001, dz = 2.24), with a mean difference of +7.6° (95% CI: 5.2-10.1). At 10 km/h, the kyphotic posture also produced significantly greater thoracolumbar flexion (12.0 ± 2.6° vs. 6.4 ± 3.1°, p < 0.001, dz = 1.85), with a mean difference of +5.6° (95% CI: 3.4-7.7). These results confirmed that the simulated kyphotic model induced a substantial and consistent increase in spinal flexion across all conditions.

**Table 1:** Comparison of spinal curvature angles between kyphotic and neutral postures across activity conditions Values are mean ± SD. All comparisons used two-tailed paired t-tests (df = 9). Δ denotes Kyphotic − Neutral. Effect size is Cohen’s dz. Significant differences are indicated by asterisks (*p < 0.05, **p < 0.01, ***p < 0.001).

Variable (unit)	Speed	Neutral	Kyphotic	t (9)	p-value		Effect size Cohen’s dz	Δ (K-N) with 95% CI
Spinal curvature (°)	Standing	6.0 ± 2.0	16.3 ± 5.0	6.38	<0.001	***	2.02	10.3 [6.6, 13.9]
	4 km/h	6.4 ± 2.2	14.0 ± 2.1	7.09	<0.001	***	2.24	7.6 [5.2, 10.1]
	10 km/h	6.4 ± 3.1	12.0 ± 2.6	5.85	<0.001	***	1.85	5.6 [3.4, 7.7]

Respiratory outcomes

The between-posture differences in respiratory parameters at each activity level are summarized in Table 2. At rest, the kyphotic posture resulted in a significantly higher RR than the neutral posture (17.5 ± 4.2 vs. 15.6 ± 3.3 breaths/min, p = 0.042, dz = 0.75), with a mean difference of +1.86 (95% CI: 0.08-3.64). ETCO₂ was higher in the kyphotic posture (4.9 ± 0.2 vs. 4.5 ± 0.1 kPa, p < 0.001, dz = 2.35), with a mean difference of +0.39 kPa (95% CI: 0.27-0.51), whereas ETO₂ was lower (15.0 ± 0.2 vs. 15.3 ± 0.4 kPa, p = 0.017, dz = −0.92; mean difference −0.39 kPa, 95% CI: −0.69 to −0.09). VD/VT was also higher in the kyphotic posture (0.37 ± 0.04 vs. 0.35 ± 0.03, unitless; p = 0.042, dz = 0.75), with a mean difference of +0.02 (95% CI: 0.00-0.04), suggesting a relative increase in dead-space ventilation at baseline. In contrast, VO₂ and VO₂/W at rest showed no significant differences between the postures (both p > 0.6).

**Table 2:** Effects of simulated kyphotic posture on respiratory parameters during standing, walking (4 km/h), and running (10 km/h) Values are presented as mean ± SD for normally distributed variables and median (Q1–Q3) for non-normally distributed variables. Normality of participant-level difference scores (Kyphotic − Neutral) was assessed with the Shapiro–Wilk test. Two-tailed paired t-tests were used for normally distributed data and Wilcoxon signed-rank tests otherwise. We report the test type, test statistic (t with degrees of freedom, or W), p-value, effect size (dz for paired t-tests; rank-biserial correlation (rrb) for Wilcoxon tests), and the mean/median difference Δ (Kyphotic − Neutral) with its 95% CI (mean difference for parametric analyses; Hodges–Lehmann median difference for nonparametric analyses). **Significant differences are indicated by asterisks (*p < 0.05, **p < 0.01, ***p < 0.001). Units: ETCO₂ and ETO₂ in kPa; VE in L/min; VO₂/W in mL/kg/min; VCO₂ in mL/min; RR in breaths/min; RER and VD/VT are unitless.

Variable (unit)	Speed	Neutral	Kyphotic	Test	Test statistic	p-value		Effect size	Δ (K-N) with 95% CI
VO_2_/W	Standing	4.8 ± 0.6	4.7 ± 0.6	t	-0.45	0.664		-0.14	-0.11 [-0.66, 0.44]
(mL/kg/min)	4 km/h	12.5 ± 1.4	12.8 ± 1.4	t	0.70	0.503		0.22	0.25 [-0.56, 1.60]
	10 km/h	33.9 ± 2.5	33.9 ± 1.8	t	0.11	0.918		0.03	0.06 [-1.21, 1.33]
VO_2_	Standing	309.0 ± 22.6	304.0 ± 50.1	t	-0.32	0.762		-0.10	-5.16 [-42.46, 32.14]
(mL/min)	4 km/h	816.6 ± 84.7	832.5 ± 92.2	t	0.65	0.531		0.21	15.9 [-39.28, 71.14]
	10 km/h	2215.2 ± 245.6	2217.0 ± 217.8	t	0.05	0.962		0.02	1.79 [-82.09, 85.67]
VCO_2_	Standing	261.3 ± 24.5	256.0 ± 35.5	t	-0.48	0.645		-0.15	-5.29 [-30.42, 19.84]
(mL/min)	4 km/h	637.6 ± 54.0	697.5 ± 95.4	t	1.86	0.096		0.59	60.75 [-13.09, 134.59]
	10 km/h	2248.6 ± 242.9	2440.1 ± 165.3	t	2.47	0.036	*	0.78	191.4 [15.98, 366.88]
RER	Standing	0.85 ± 0.10	0.85 ± 0.05	t	0.13	0.940		0.04	0.00 [−0.05, 0.06]
(unitless)	4 km/h	0.78 (0.75–0.81)	0.80 (0.77–0.87)	W	6.0	0.049	*	0.63 (rrb)	0.06 [-0.01, 0.12]
	10 km/h	1.02 ± 0.06	1.11 ± 0.09	t	3.87	0.004	**	1.23	0.09 [0.04, 0.14]
RR	Standing	15.6 ± 3.3	17.5 ± 4.2	t	2.37	0.042	*	0.75	1.86 [0.08, 3.64]
(breaths/min)	4 km/h	25.7 ± 4.7	23.8 ± 4.3	t	-1.31	0.223		-0.41	-1.85 [-5.05, 1.35]
	10 km/h	36.0 ± 6.9	35.7 ± 6.8	t	-0.14	0.888		-0.05	-0.31 [-5.16, 4.54]
VE	Standing	12.2 ± 0.7	11.9 ± 1.6	t	-0.61	0.559		-0.19	-0.30 [-1.42, 0.82]
(L/min)	4 km/h	23.7 ± 3.7	24.3 ± 3.4	t	0.48	0.645		0.15	0.57 [-2.14, 3.27]
	10 km/h	65.8 ± 9.6	69.5 ± 7.1	t	1.52	0.163		0.48	3.73 [-1.82, 9.28]
ETO_2_	Standing	15.3 ± 0.4	15.0 ± 0.2	t	-2.92	0.017	*	-0.92	-0.39 [-0.69, -0.09]
(kPa)	4 km/h	13.9 ± 0.5	13.9 ± 0.5	t	-0.05	0.959		-0.02	-0.01 [-0.43, 0.41]
	10 km/h	14.5 ± 0.7	14.7 ± 0.7	t	1.41	0.192		0.45	0.21 [-0.13, 0.55]
ETCO_2_	Standing	4.5 ± 0.1	4.9 ± 0.2	t	7.41	<0.001	***	2.35	0.39 [0.27, 0.51]
(kPa)	4 km/h	5.3 ± 0.3	5.5 ± 0.4	t	5.58	<0.001	***	1.77	0.32 [0.19, 0.45]
	10 km/h	6.0 ± 0.5	6.1 ± 0.5	t	1.48	0.173		0.47	0.14 [-0.07, 0.35]
VE/VO_2_	Standing	40.4 ± 3.5	40.0 ± 4.5	t	0.28	0.788		0.09	0.42 [-3.01, 3.85]
(unitless)	4 km/h	29.1 ± 3.7	29.3 ± 3.7	t	-0.18	0.859		-0.06	-0.23 [-3.07, 2.61]
	10 km/h	29.8 ± 3.8	31.7 ± 5.0	t	1.71	0.121		0.54	1.91 [-0.61, 4.43]
VE/VCO_2_	Standing	47.8 ± 3.9	47.4 ± 6.0	t	-0.38	0.723		-0.12	-0.38 [-2.73, 1.97]
(unitless)	4 km/h	37.2 ± 4.0	35.0 ± 3.3	t	-2.17	0.058		-0.69	-2.21 [-4.51, 0.09]
	10 km/h	29.3 ± 2.8	28.5 ± 2.6	t	-1.09	0.305		-0.34	-0.77 [-2.37, 0.83]
VD/VT	Standing	0.35 ± 0.03	0.37 ± 0.04	t	2.37	0.042	*	0.75	0.02 [0.00, 0.04]
(unitless)	4 km/h	0.32 ± 0.03	0.32 ± 0.02	t	-0.67	0.519		-0.21	0.00 [-0.03, 0.02]
	10 km/h	0.26 ± 0.02	0.25 ± 0.01	t	-0.32	0.758		-0.10	0.00 [-0.02, 0.01]

At 4 km/h, the respiratory exchange ratio (RER, unitless) was higher under the kyphotic posture (0.80 [0.77-0.87] vs. 0.78 [0.75-0.81]), reaching statistical significance (Wilcoxon W = 6.0, p = 0.049, rrb = 0.63; mean difference +0.06, 95% CI: −0.01-0.12). ETCO₂ remained elevated in the kyphotic posture (5.5 ± 0.4 vs. 5.3 ± 0.3 kPa, p < 0.001, dz = 1.77), with a mean difference of +0.32 kPa (95% CI: 0.19-0.45). VO₂ and VO₂/W did not differ significantly between the postures (p > 0.5). VCO₂ showed a non-significant trend toward higher values in the kyphotic posture (697.5 ± 95.4 vs. 637.6 ± 54.0 mL/min, p = 0.096, dz = 0.59). VE/VCO₂ also tended to be lower in the kyphotic posture (35.0 ± 3.3 vs. 37.2 ± 4.0, p = 0.058), although the difference was not statistically significant. VD/VT and ETO₂ did not differ significantly between postures.

At 10 km/h, the kyphotic posture increased the ventilatory burden, with a significantly higher CO₂ output (VCO₂: 2440.1 ± 165.3 vs. 2248.6 ± 242.9 mL/min, p = 0.036, dz = 0.78), yielding a mean difference of +191.4 mL/min (95% CI: 15.98-366.88). RER was also higher (1.11 ± 0.09 vs. 1.02 ± 0.06, p = 0.004, dz = 1.23), with a mean difference of +0.09 (95% CI: 0.04-0.14). VE showed a non-significant trend toward higher values in the kyphotic posture (69.5 ± 7.1 vs. 65.8 ± 9.6 L/min, p = 0.163, dz = 0.48), with a mean difference of +3.73 L/min (95% CI: −1.82-9.28). VO₂ and VO₂/W showed no significant posture-related differences (p > 0.9). RR and VD/VT were also comparable between the conditions. ETO₂ at this workload did not differ significantly (14.7 ± 0.7 vs. 14.5 ± 0.7 kPa, p = 0.192).

Taken together, these findings, supported by moderate-to-large effect sizes, with CIs generally consistent with posture-related differences, indicate that kyphotic posture increases respiratory demand, particularly during high-intensity exercise. Although some variables (e.g., VE at 10 km/h) did not reach statistical significance, the observed effect sizes suggested a potentially meaningful ventilatory burden under these conditions.

## Discussion

Unlike previous studies [1-4] that focused only on pulmonary function in static postures, our study adds experimental evidence that kyphotic alignment impairs ventilatory efficiency during dynamic exercises such as walking and running. By evaluating respiratory parameters in healthy adults across standing, walking, and running conditions, we found that kyphotic posture consistently altered respiratory dynamics, particularly at rest and during high-intensity exercises. These results provide novel evidence that spinal alignment directly influences real-time respiratory performance during functional activity. Importantly, these results suggest that even mild postural deviations can impose measurable respiratory burdens on healthy individuals.

Resting condition

At rest, participants in the kyphotic posture showed significantly higher RR and ETCO₂, along with lower ETO₂ and higher VD/VT. These findings suggest that kyphotic alignment reduces thoracic expansion and diaphragmatic excursion, thereby limiting VT. The flexed thoracic position restricts anteroposterior rib cage expansion and reduces caudal displacement of the diaphragm, which, in turn, diminishes the generation of inspiratory negative pressure. As a result, participants adopt rapid, shallow breathing, which increases respiratory frequency and dead-space ventilation, mirroring the patterns observed in older adults and individuals with neuromuscular or respiratory impairments [10-12]. This mechanical restriction shifts the ventilatory strategy toward higher-frequency breathing with reduced alveolar gas-exchange efficiency and increased reliance on accessory inspiratory muscles [11,13]. These patterns (ETCO₂↑/ETO₂↓, VD/VT↑) are consistent with reduced alveolar ventilation relative to metabolic production secondary to lower VT and a higher dead-space fraction in the kyphotic posture.

Moderate-intensity exercise (4 km/h)

During walking at 4 km/h, ETCO₂ remained elevated in the kyphotic posture, indicating persistent ventilatory inefficiency despite maintained VO₂. This persistence implies that the mechanical constraints on thoracic expansion and diaphragmatic motion remain operative during moderate workloads [11]. With the diaphragm’s contribution reduced, reliance on accessory respiratory muscles, such as the sternocleidomastoid and scalenes, increases, raising the energetic cost of breathing. Although the statistical differences in VE/VCO₂ were not significant at this intensity, the consistent trend toward greater efficiency in the neutral posture suggests that posture correction can mitigate unnecessary respiratory load during submaximal activity. The tendency toward a lower VE/VCO₂ ratio suggests reduced ventilatory efficiency, plausibly driven by greater accessory muscle recruitment and suboptimal thoracoabdominal mechanics.

High-intensity exercise (10 km/h)

At 10 km/h, the kyphotic posture produced significantly higher CO₂ output (VCO₂) and RER, with a non-significant trend toward higher VE than the neutral posture. Together with RER > 1.0 at this workload, these findings indicate an earlier shift toward anaerobic metabolism and heightened respiratory compensation. Under kyphotic alignment, the diaphragm must simultaneously contribute to trunk stabilization and ventilation, reducing its efficiency for either role [13,14].

Consequently, ventilation increases without a commensurate increase in oxygen uptake, elevating the oxygen cost of breathing and potentially limiting locomotor O₂ delivery. Over sustained efforts, this pattern may accelerate fatigue onset and impair performance in both healthy and clinical populations.

This pattern is consistent with thoracoabdominal mechanical constraints that could increase the oxygen cost of breathing, in line with the higher ETCO₂ at rest, higher RER during walking and running, and higher VCO₂ during running, despite similar VO₂; however, because diaphragmatic mechanics were not directly imaged, mechanistic inferences remain provisional.

Clinical and practical implications

This study suggests that kyphotic posture during high-intensity exercise may accelerate respiratory muscle fatigue and impair aerobic endurance in healthy young adults. Such effects are relevant not only for older adults and individuals with compromised respiratory function but also for endurance athletes, including runners and swimmers, for whom ventilatory efficiency is critical for performance and resistance to fatigue.

Interventions aimed at improving thoracic mobility and spinal alignment, such as thoracic extension exercises or posture-focused physiotherapy, have the potential to reduce kyphotic curvature and enhance pulmonary function [5,6,15-18]. Manual therapy approaches, including thoracic spinal manipulation, have also demonstrated immediate improvements in pulmonary function among sedentary young adults [5], suggesting that mobility-oriented strategies could complement postural training in optimizing respiratory performance [19-21]. Given their low cost and ease of implementation, these interventions can be applied in both clinical rehabilitation and athletic training to reduce postural-induced ventilatory burden.

The present findings indicate that even in healthy adults, transient kyphotic posture can significantly impair respiratory efficiency during exercise. For athletes, even small reductions in ventilatory efficiency could accumulate and negatively influence endurance capacity and competitive performance. In populations with existing respiratory compromise, such as individuals with neuromuscular disorders or postural deformities, the impact may be more pronounced, potentially limiting daily activity tolerance and rehabilitation [22,23]. These results emphasize the importance of early postural assessment and intervention strategies, not only to optimize athletic performance but also to support respiratory rehabilitation in clinical settings.

Furthermore, early detection of posture-induced respiratory inefficiency in healthy adults could serve as a marker for future functional decline, supporting preventive postural training before the development of structural deformities. For endurance athletes, a neutral posture during locomotion may optimize ventilatory efficiency and metabolic economy, thereby delaying fatigue and supporting long-term performance. Similarly, in elderly populations or patients with respiratory impairments, maintaining proper spinal alignment may alleviate the ventilatory load, enhance gas exchange, and improve exercise tolerance.

In summary, our findings suggest that even in healthy young adults, kyphotic posture compromises ventilatory efficiency during walking and running, suggesting that posture plays a fundamental role in sustaining optimal respiratory mechanics during dynamic exercise. These findings highlight the potential value of posture-focused intervention. Strategies such as thoracic mobility exercises, posture correction programs, and respiratory physiotherapy can reduce ventilatory inefficiency and enhance exercise tolerance in older populations and individuals with respiratory impairments. In athletic contexts, incorporating postural training into performance programs may improve ventilatory efficiency, metabolic economy, and endurance capacity. Notably, these posture-related differences were accompanied by moderate-to-large effect sizes, suggesting that the observed impairments in ventilatory efficiency were not only statistically significant but also physiologically meaningful.

Limitations

This study has several limitations. First, the sample was small and homogeneous (n = 10 healthy young men), which limits generalizability to women, older adults, and clinical populations and reduces statistical power. Second, the kyphotic posture was experimentally simulated rather than structural, so external validity to clinical kyphosis is uncertain. Third, ventilatory mechanics were not directly assessed (e.g., diaphragmatic imaging or respiratory muscle EMG), constraining mechanistic inference. Fourth, VO₂max was estimated via a submaximal cycling protocol rather than measured directly, which may limit characterization of absolute fitness.

These constraints warrant cautious interpretation. Future studies should include larger, sex-balanced, and age-diverse cohorts, as well as individuals with structural kyphosis, and incorporate objective tools for posture standardization.

## Conclusions

A kyphotic alignment was linked to a measurable increase in ventilatory burden at rest and during running-higher ETCO₂ (rest), higher RER (walking and running), and higher VCO₂ (running), despite similar VO₂. Given the small, homogeneous sample and the simulated posture model, these results should be considered preliminary; replication in larger, sex- and age-diverse cohorts, including individuals with structural kyphosis, is needed. If reproduced, the findings may inform posture-focused strategies (e.g., thoracic mobility and postural training) in rehabilitation and sports.
